# Cross-sectional community-based study of the socio-demographic factors associated with the prevalence of dengue in the eastern part of Sudan in 2011

**DOI:** 10.1186/s12889-015-1913-0

**Published:** 2015-06-18

**Authors:** Mohammed A. Soghaier, Sayed Himatt, Kamal ElDin Osman, Somia I. Okoued, Osama E. Seidahmed, Mark E. Beatty, Khalifa Elmusharaf, Jeahan Khogali, Nijood H. Shingrai, Mutasim M. Elmangory

**Affiliations:** Epidemiology and Zoonotic Diseases, Federal Ministry of Health, Osman Digna Street with Nile Avenue, PO Box 303, 1111 Khartoum, Sudan; Present Address: Supreme Council of Health, Doha, Qatar; Community Department, Al Ribat University, Faculty of Medicine, Khartoum, Sudan; Tiabah University, College of Medicine, Family and Community Medicine, Riyadh, Saudi Arabia; Emergency and Humanitarian Action, Federal Ministry of Health, Khartoum, Sudan; Department of Medical Entomology, National Public Health Laboratory, Khartoum, Sudan; Biogen Idec, Cambridge, MA USA; Department of Epidemiology and Public Health Medicine, The Royal College of Surgeons in Ireland, Manama, Bahrain; Epidemiology and Zoonotic Diseases, Kassala State Ministry of Health, Kassala, Sudan; Sudan National Public Health Laboratory, Khartoum, Sudan

**Keywords:** Sero-prevalence, Dengue, Social factors, Capture ELISA, Eastern Sudan

## Abstract

**Background:**

Dengue is caused by an arthropod-borne flavivirus. Infection can be either primary or secondary based on serology, with each stage of the disease characterized by specific serological conversion and antibody formation. Further study is needed to fully identify the factors associated with and predisposing to dengue infection. The objective of this study was to identify socio-demographic factors associated with the prevalence of dengue serotypes in Kassala State in the eastern part of Sudan in 2011.

**Methods:**

This was a cross-sectional community-based study with 530 participants who were randomly selected through multi-stage cluster sampling. Dengue serotype prevalence was determined using capture Enzyme-linked immunosorbent assay (ELISA). ELISA IgG. A multivariate logistic regression model was designed to measure the strength of associations between socio-demographic factors and dengue serotype prevalence. All participants who tested negative for dengue were used as the statistical reference group.

**Results:**

From this study, the prevalence of dengue in Kassala was estimated to be 9.4 % (95 % CI: 7.1–12.3). Lack of knowledge about dengue fever disease (OR 2.8, 95 % CI: 1.24–6.53) and a household density of more than 3 people per room (OR 2.1, 95 % CI: 1.06–4.09) were the most important factors associated with dengue infection among the study population.

**Conclusions:**

Community-oriented interventions are needed to modify existing social behaviors to reduce the risk of dengue in the eastern part of Sudan. Additional studies are also required in this field.

## Background

Dengue is caused by an arthropod-borne flavivirus [1], and is currently endemic in several countries worldwide [2]. It is estimated that there are 390 million (95 % credible interval 284–528) dengue infections per year, of which 96 million (67–136) produce symptoms of varying levels of severity) [3]. Dengue virus has four known serotypes. Homotypic immunity (specific to the same serotype) is developed after infection with any one of the four serotypes, and is believed to be lifelong. There is also evidence of heterotypic immunity to all serotypes which decreases about 6 months after infection.

Improvements in laboratory tests allow dengue infections to be classified as either a primary or secondary infection, and either a recent or old infection. Primary dengue infection is defined as the first experience of a human host with dengue virus and can manifest in one of three clinical forms. In the majority of patients, it presents as an undifferentiated febrile illness, with cold-like symptoms. Other patients develop severe body pain and high fever, which is classic dengue fever. The remaining patients experience a severe form of dengue presenting as dengue hemorrhagic fever (DHF) or dengue shock syndrome (DSS) [4].

Secondary dengue is defined as contracting dengue virus after an initial exposure and symptoms, and carries an enhanced risk of DHF/DSS/severe dengue fever due to cross-immunity reactions. The eastern part of Sudan has experienced repeated outbreaks of dengue during the last couple of years particularly from 2005 onward. The areas most affected are Red Sea and Kassala states, which have dengue virus types 1 and 3 in circulation [5, 6]. Moreover, DEN-1, DEN-2, and DEN-3 have been confirmed in the port city of Jeddah just across the Red Sea from Kassala, and with connections to the eastern part of the Sudan [7].

While some factors associated with dengue infections are well understood, others require further investigation. For example, age has an effect on dengue epidemiology, as the likelihood of being seropositive for dengue increases with age because of increased exposure to the viremic vector. Severe dengue virus infection causes higher fatality in very young and very old patients. Military-aged adults from dengue endemic countries are not usually at risk for developing severe dengue disease; however, studies of traveling populations from non-endemic US or Europe have demonstrated that similar-aged naïve populations can succumb to severe dengue disease [8]. Population movement or travel is also considered to be a risk factor because this increases the chance of being exposed to different dengue serotypes from those in the primary residence, and this is sometimes associated with the severe form of the disease, DHF/DSS. Around 80 % of patients with the severe form of dengue have secondary dengue. The odds of developing DHF on primary exposure to dengue is 0.5 %, which increases to around 5 % upon secondary exposure to a different serotype [9]. In addition, viremic travelers may contribute to spreading the disease and propagating disease-related outbreaks, particularly in areas that have competent vectors to transmit dengue. In addition, high dengue viremia titers correlate with a greater probability of DHF/DSS [10]. Knowledge of dengue and about protective measures to prevent infection are considerable tools to prevent both primary and secondary dengue infection [11].

Dengue is an endemic disease in the eastern part of Sudan, with repeated dengue outbreaks during recent years in 2005, 2008 and 2010. The aim of this study was to assess and define social and demographic risk factors for dengue infections in this region.

## Methods

### Study design

This analytical cross-sectional community-based study was performed in Kassala. Kassala is a border state of Sudan, with Eritrea and Ethiopia to the east and national borders with Red Sea state to the north, Khartoum, River Nile, and Gadarif states to the west and south west. The total population of Kassala over the age of 5 years was considered as a source population for this study. Exclusion criteria were those not resident in the state for the previous 6 months, patients with chronic debilitating diseases such as cancers, and those taking immunosuppressant medications.

### Sampling technique

A multi-stage cluster sampling technique [12] was used for this study. First, 27 out of the 97 popular administrative units in Kassala were randomly selected for sampling using the probability proportionate to size technique [13]. Second, households were selected using a systematic, random sampling technique. Within the household, one participant was selected from the eligible population who were present at the time of the data collector’s visit using a simple random sampling approach (random generating table).

### Sample size to assess risk factors

A sample of 530 participants was needed to estimate the prevalence of dengue serotypes, and its association with socio-demographic factors with 90 % power and a 95 % confidence level, assuming the measure of association is greater than the null value (OR >1) and a design effect of 2 to adjust for the sampling technique. The formula for the calculation of sample size was applied using OpenEpi software [14].

### Data collection and statistical analysis

A structured questionnaire was used by trained data collectors to collect socio-demographic variables. Venous blood samples were collected for dengue serotyping. A multivariate logistic regression model was designed to quantify the strength of association between study outcome and anticipated risk factors using SPSS-19 and STATA-12 software.

### Laboratory analysis and outcome determination

Blood samples were analyzed using Panbio ELISA kits (DF IgG capture) [15]. According to the manufacturer “the Panbio Dengue IgG Capture ELISA” is for the qualitative presumptive detection of elevated IgG antibodies to dengue virus (serotypes 1–4). The IgG Capture ELISA has a serological sensitivity of 97.9 % (CI: 92.5–99.7 %) and specificity (negatives) of 100 % (CI: 96.6–100.0 %) [16].

### Ethical considerations

Ethical approval was granted from the ethical review committee of both the Sudan Medical Specialization Board in Khartoum and Kassala State Ministry of Health. Signed informed consent was obtained from all surveyed participants. Participation in the survey was voluntary; neither gifts nor financial incentives were offered.

## Results

Of the selected participants, 491 (91 %) agreed to take part, completed the administered questionnaire and gave blood sample. Overall, 60 % of participants were male and the mean age of the participants was 36.6 years, ranging from 13 to 85 years. Baseline characteristics of the study participants are summarized in Table 1. The prevalence of serotyped dengue was estimated to be 9.4 % (95 % CI: 7.1–12.3). The statistical analysis considered all negative samples as the study control group.

**Table 1:** Main individual characteristic of study participants, Kassala 2011

Characteristics	Frequency	Percent (%)
Result of the HH interview (*n* = 540)		
Completed	491	91
Refuse	49	9
Age groups (*n* = 524)		
< 25 years	106	20
25–35 years	193	37
36–45 years	100	19
> 45 years	125	24
Mean age (95 % CI)	36.59	(35.4–37.8)
Age range (in years) Max – Min (SD)	13–85	(14)
Sex of the interviewee (*n* = 525)		
Male	210	40
Female	315	60
Highest level of school (*n* = 493)		
Never attended school	88	18
Informal education	59	12
Primary school	162	33
Intermediate school	31	6
Secondary school	107	22
College/certificate	42	9
Higher education	4	1
Relation of the respondent to household head (*n* = 528)		
Household head	147	28
Husband/wife	195	37
Son/daughter	130	25
Father/mother	20	4
Brother/sister	18	3
Others	18	3

In the multivariate analysis, people who had never heard about dengue (adjusted OR 2.8, 95 % CI: 1.24–6.53), and households with a density of more than 3 people per room (adjusted OR 2.1, 95 % CI: 1.06–4.09) were found to be most likely to be dengue seropositive. The risk of developing dengue infection among people who have never heard about the disease is almost 3 times the risk of the disease among those who have heard about it, and the risk is 2 times more among the residents of crowded households compared with those living in less crowded dwellings. All other factors studied were found to have insignificant statistical association with the study outcome. The multivariate analysis model of factors associated with dengue sero-prevalence is summarized in Table 2.

**Table 2:** Logistic regression analysis for factors associated with dengue sero-prevalence among study Population, Kassala Locality 2011

Characteristic	Positive/tested	Multivariate analysis		
		Adjusted OR	95 % CI	P value
Age				
35 years or less	28/281	1.17	0.55–2.5	0.69
> 35 years	18/206			
Sex				
Males	29/288	1.55	0.74–3.25	0.24
Females	17/199			
Education level				
No formal education	16/144	0.84	0.38–1.86	0.67
Formal education	30/344			
Permanent residence				
Live outside Kassala	1/12	1.31	0.14–11.82	0.81
Live in Kassala	45/472			
Travel to Red Sea in the last 6 months				
Yes	4/42	1.36	0.34–5.52	0.66
No	42/442			
Had a guest from Red Sea in the last 6 months				
Yes	9/129	0.86	0.35–2.08	0.73
No	37/354			
HH member travel to Red Sea in the last 6 months				
Yes	4/92	0.37	0.10–1.38	0.14
No	42/392			
Had a yellow fever vaccine before				
Yes	4/36	1.66	0.48–5.78	0.42
No	42/446			
Source of drinking water		1.17	0.55–2.5	0.69
Not piped water	6/47	1.35	0.49–3.72	0.56
Piped water	40/438			
Screens in the windows				
No intact screen	40/411	0.84	0.31–2.25	0.73
Intact screen	6/70			
Using Bed net				
Not using bed net	23/241	1.08	0.54–2.18	0.82
Using bed net	23/242			
Never heard about dengue				
No	27/197	2.84	1.24–6.53	0.014
Yes	19/289			
Household density				
> 3	24/178	2.08	1.06–4.09	0.034
3 or less	22/311			
Household inspection for adult Ae.aegypti				
Positive	11/116	0.55	0.19–1.61	0.28
Negative	35/373			
Household inspection for Pupa Ae.aegypti				
Positive	8/53	2.54	0.71–9.08	0.15
Negative	38/436			

## Discussion

In this study, people who had never heard about dengue fever were found to be more at risk of getting dengue infection, with an adjusted OR of 2.8 (95 % CI: 1.24–6.53). This could be explained by a lack of knowledge regarding susceptibility to the disease and the appropriate protective measures. This lack of knowledge is directly exposing participants to a greater risk of contracting the disease compared with others who are well aware about dengue disease dynamics and take preventive measures [17]. Population knowledge is a vital social determinant of dengue disease epidemiology because community participation is believed to be a key element for current recommended dengue control and prevention strategies worldwide [18]. Household density, which refers to the number of people per room, was found to be a statistically significant predictor of dengue sero-prevalence amongst the study population, with an odds ratio of 2.1 (95 % CI: 1.06–4.09). This indicates that residents of a household with a density of greater than 3 people per room are more likely to get dengue infection compared with those who live in less crowded accommodation. This finding supports existing beliefs regarding dengue in different parts of the world-that human host density is considered one of the important social determinants of dengue infections. Furthermore, small household size is being confounded by overall human density in other studies [19, 20]. This could be attributed to increased exposure of susceptible people to the dengue vector in overcrowded households. The responsible vector, the Aedes mosquito, feeds on multiple humans per day, facilitating transmission of the virus in an efficient manner [11]. In addition, household density itself is always associated with poverty and low socioeconomic status, particularly in developing countries, and this is another social predictor for dengue infections [19]. Age of participants in the current study was not statistically associated with dengue fever seropositivity (OR 1.17 (95 % CI: 0.55–2.5)). This is in contrast to a published study that found a higher prevalence of dengue seropositivity with increasing age, which may be due to the increased likelihood of being exposed to a mosquito bite and becoming dengue seropositive over a life span [19]. In this study, sex did not show a significant association with dengue infection (OR 1.55 (95 % CI: 0.74–3.25)). A similar study, conducted in the southern part of Sudan, had concluded females were at higher risk of developing dengue, which was explained by the fact that in such conservative communities, females usually spend the majority of their time at home and the dengue vector breeds inside homes [19, 21]. Any association between dengue susceptibility and sex is debatable, as there are contradictions between the available studies to date [22, 23].

Kassala is an urban area located on the highway connecting Khartoum, the capital of Sudan, with the main sea harbor of the country, Port-Sudan city on the Red Sea coast with large population movements and social connections between Kassala and Red Sea state. Families from Port-Sudan usually spend the hot humid months of summer in Kassala taking advantage of more pleasant weather during that season. Port-Sudan has been known as an endemic area for dengue since the 1980s, with repeated dengue outbreaks in recent years particularly since 2005 onward [5, 6, 24–27]. It was assumed that dengue disease was imported from Port-Sudan to Kassala; however, in this study, travel of a participant or one of his household members to Red Sea state in the last 6 months was not statistically significantly associated with dengue prevalence in Kassala. Movement of dengue viremic patients is believed to facilitate introduction of new dengue serotypes to other areas and sustain the circle of transmission and propagation [19, 28]. Having a guest from Red Sea, storing water at home, the absence of window screens, the presence of Aedes aegypti pupa and adult mosquitos in the house or a history of yellow fever vaccination were all found not to be statistically associated with dengue infection in this study.

Although this is the first attempt to study factors associated with dengue sero-conversion in Kassala, the current study has its limitations, including the fact that it was carried out among community participants regardless of their symptomatic state; this makes it difficult to differentiate between primary and secondary dengue infections. Sometimes IgG levels wane below the IgG ELISA cut-off and this leads to missing cases (both primary and secondary) several years following exposure. Monotypic and polytypic plaque reduction neutralization tests (PRNT) responses could be assessed in the future for more accurate estimation of overall prevalence. Moreover, there is a possibility of cross reaction with other favivirus antibodies that may be circulating in the study area, particularly Yellow fever and Zika viruses that have been documented in other parts of the country, although there are no specific studies conducted in Kassala to date.

## Conclusion

People who had never heard of dengue and those living in a household density of more than 3 people per room were those most likely to test seropositive for dengue in Kassala in 2011. Intensive social interventions are required to increase community awareness of dengue to minimize the risk of the severe complications of this infection. Further detailed studies and operational research with more advanced epidemiological methodologies are important to fill the existing knowledge gaps in terms of dengue dynamics in the region.
